# Results of an experimental study of subgingival cleaning effectiveness in the furcation area

**DOI:** 10.1186/s12903-021-01736-4

**Published:** 2021-08-02

**Authors:** Miriam Seidel, Hannah Borenius, Susanne Schorr, David Christofzik, Christian Graetz

**Affiliations:** Clinic of Conservative Dentistry and Periodontology, University of Kiel, Kiel, Germany

**Keywords:** Scaling and root planning, Nonsurgical periodontal debridement, Furcation defects

## Abstract

**Background:**

Sufficient biofilm removal in the furcation area (FA) is a major challenge in the clinical practice of supportive periodontal therapy. The aim of the present experimental study was to simulate subgingival cleaning of the FA using a powered scaler (sonic scaler (AIR), ultrasonic scaler (US)) for conventional mechanical debridement versus two air polishing with nonabrasive powder (LAPA-1: glycine powder, LAPA-2: erythritol powder) and different nozzles for supra-/subgingival cleaning for each device.

**Methods:**

Seven trained and calibrated operators with ≥ 2 years each of professional experience in treating periodontitis used the instruments to clean 3D-printed replicas of six molars with through-and-through FA (four 3-rooted and two 2-rooted teeth) in a manikin head. AIR and US were used in the control group; air polishing instruments were used in the test group. For reproducible evaluation, the test teeth were separated vertically into two or three parts, illuminated with ultraviolet light, photographed and evaluated planimetrically. Treatment time (TrT, in s) and relative cleaning efficacy (RCE, in %) were measured.

**Results:**

Overall, 3-rooted molars (RCE in the entire FA, 23.19 ± 20.98%) could be cleaned significantly less effectively than 2-rooted molars (53.04 ± 28.45%, *p* < 0.001), regardless of the instrument used. In the cleaning of the entire FA, significantly higher RCE values were achieved with conventional mechanical debridement (AIR/US: 46.04 ± 25.96%/39.63 ± 22.02%; AIR vs. US: *p* > 0.05) than with air polishing (LAPA-1/LAPA-2: 34.06 ± 29.48%/17.09 ± 18.85%; LAPA-1 vs. LAPA-2: *p* < 0.001) regardless of whether a supra- or subgingival cleaning nozzle used (*p* < 0.001). Only LAPA-1 with a subgingival nozzle showed RCE values comparable to those of US (41.07 ± 28.95% vs. 39.63 ± 22.02%, *p* > 0.05). TrT was longest for US (299.40 ± 120.69 s) and shortest for LAPA-1 with a supragingival nozzle (129.67 ± 60.92 s, *p* < 0.001).

**Conclusions:**

All of the examined instruments were effective to some degree in removing the simulated biofilm from the FA, but they differed substantially in cleaning efficacy. Only one air polishing device (LAPA-1) with a rigid subgingival nozzle was able to achieve RCE values similar to those of US. The current investigation confirmed that conventional mechanical debridement with powered scalers were most effective, but treatment took longer with these devices than air polishing.

## Background

Periodontitis is described as a multifactorial inflammatory disease associated with dysbiotic biofilms and characterized by progressive destruction of the periodontium [1]. Adequate active periodontal therapy restores the biocompatibility of the previously diseased root surfaces [2, 3], allowing reattachment of adjacent tissues [4–7]. However, successful active periodontal therapy must be followed by regular appointments for professional mechanical biofilm removal as part of supportive periodontal therapy (SPT) [8]. Accordingly, several visits for SPT are necessary in a patient’s lifetime to prevent further periodontal inflammation, and professional mechanical biofilm removal should be performed with special attention to teeth with exposed root surfaces or residual pockets [9]. The use of a hand or powered scaler (SC) to remove mineralized and nonmineralized biofilms is the most widely accepted method of conventional mechanical debridement [10, 11], while air polishing (AP) is preferred for nonmineralized biofilms [12]. In particular, low-abrasiveness powder air polishing (LAPA) is recognized as a minimally invasive tool for the management of biofilms colonizing tooth and root surfaces [12], as effective biofilm removal can be achieved while preserving the integrity of the root surface and soft tissue [13]. Different low-abrasiveness powders, e.g., glycine and erythritol, have been demonstrated to provide a fast and reliable method for removing subgingival biofilms, requiring a high level of comfort for both the patient and the operator [11, 14]. Petersilka et al. [11] found that LAPA with glycine was equivalent to solely subgingival regarding the number of sites with stable pocket probing depths (PPDs) and the biofilm index in long-term SPT. However, for molars with furcation involvement (FI), the authors noted that these root sites were not as treatable as other sites and recommended SC. Furthermore, they noted a trend toward deterioration of the FI status in the LAPA group and indicated the use of conventional “showerhead”-like air polishing nozzles in molar furcations as a possible reason [11]. Another recent study by Ulvik et al. [15] investigated the clinical parameters of erythritol air polishing versus curette/ultrasonic cleaning in the treatment of mandibular molars with grade II furcation, revealing that, despite the use of a special nozzle for air polishing, there was a significant difference in clinical attachment level in favor of hand or powered instruments after 6 months. Nevertheless, the reason may not yet be fully understood. Therefore, the aim of this experimental study was to evaluate the in vitro effectiveness of subgingival biofilm removal with two SC devices, a sonic scaler (AIR) and an ultrasonic scaler (US), versus two different AP devices (LAPA-1 and LAPA-2) with and without subgingival nozzles in molars with through-and-through FI [16].

## Methods

Before the study, all seven operators were asked to participate, and after giving their written consent, they were included. All operators were employees of the Department of Periodontology, Christian-Albrechts-University Kiel, and they had three to twenty-two years of professional experience. They had completed the same training program, including lectures on the applicable theoretical information according to our clinical guidelines and the manufacturer’s guidelines, as well as practical sessions before the test. Additionally, all operators were clinically calibrated for application pressure (< 1 N for AIR and US) during training sessions, but no measurements of root surface destruction or roughness were made during testing.

The frequency of the instruments and the test teeth as well as the order of the tested instruments were randomized (Microsoft Excel 16, Microsoft Corporation, One Microsoft Way Redmond, WA, USA) for each operator to exclude any influences of laterality or training effects. Each operator used all instruments in a randomized order on the same day. Operators were asked to instrument the test teeth until they subjectively judged that they had maximally eliminated the simulated biofilm. The time taken to treat one tooth was recorded for each instrument and each operator.

### Experimental setup

All operators had the same setup and instruments for root surface instrumentation: (1) the LAPA-1 air polishing device (LM-ProPower, LM-Instruments Oy, Parainen, Finland) on the middle level with a supragingival nozzle (LM-Supra A nozzle, universal, LM-Instruments Oy, Parainen, Finland) and (2) with a subgingival nozzle (LM-Sub A nozzle, LM-Instruments Oy, Parainen, Finland); (3) the LAPA-2 air polishing device (AIRFLOW PROPHYLAXIS MASTER, EMS, Nyon, Switzerland) on the middle level with a supragingival nozzle (AIRFLOW handpiece, EMS, Nyon, Switzerland) and (4) with a subgingival nozzle (PERIOFLOW handpiece, EMS, Nyon, Switzerland); (5) a US (Proxeo ultra, W&H, Bürmoos, Austria) on the middle level (water cooling: 30 mL/min) equipped with a straight slimline tip with a round cross-section (1P, W&H, Bürmoos, Austria); and (6) an AIR (SONICflex 2003 L, KaVo Dental, Biberach, Deutschland) on the middle level (water cooling: 30 mL/min) equipped with a straight slimline tip with a round cross-section (paro Nr.60, KaVo Dental, Biberach, Deutschland). For US and AIR, only new instrument tips were used for each operator. LAPA-1 was utilized with glycine powder (LM-Glycine Neutral, LM-Instruments Oy, Parainen, Finland) with a particle size of 25 μm (LAPA-2: erythritol powder with a particle size of 14 µm, AIR-FLOW PLUS powder, EMS, Nyon, Switzerland).

### Manikin heads and test teeth

**Fig. 1:**
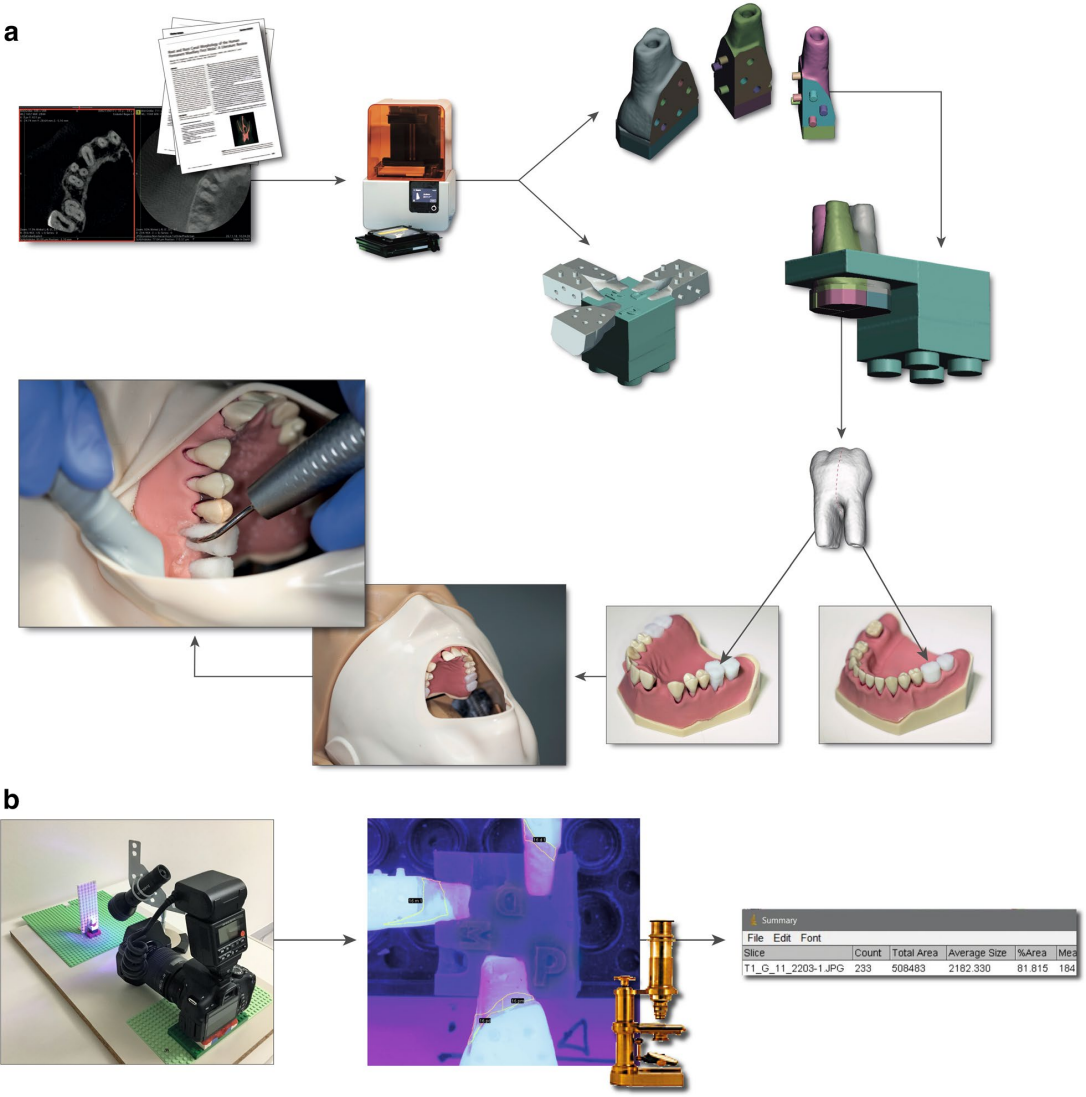
Graphical representation of the test preparation, the test run and the evaluation. **a** Based on data from the literature and with the help of three-dimensional radiographs, datasets were created to produce anatomical models of teeth with furcation involvement using a 3D printer. A special anatomical design for the inner crown was used to create surfaces according to the lock-and-key principle so that each root of each tooth could be evaluated in a standardized position. Coated, 3D-printed anatomical models of the 3-rooted test teeth at positions 17, 16, 26 and 27 and of the 2-rooted test teeth at positions 37 and 36 were inserted into the modified periodontitis model (Frasaco, Tettnang, Germany) and fixed in a manikin head with screws from the apical direction. Subsequently, the furcation areas of all 6 molars were treated with the various instruments by 7 operators. **b** After the 6 molars in the model were cleaned by the various operators, the teeth were removed from the model, and all 3-rooted test teeth were divided vertically into three parts (2-rooted test teeth into two parts). The parts were integrated into an individual unit for each of 6 teeth (made of LEGO, Lego GmbH, Grasbrunn, Germany), with the help of the specially designed inner crown surface, thus enabling evaluation in the same position. For the evaluation, the teeth were photographed under ultraviolet light and analyzed in ImageJ software (ImageJ, NIH, Bethesda, MD, USA)

All tests were performed on a manikin head with modified periodontitis models (Frasaco, Tettnang, Germany) that exhibited pronounced periodontitis with moderate to advanced horizontal bone loss and isolated and deep vertical pockets, resulting in varying difficulty in instrumenting the teeth in terms of both anatomy and accessibility (Fig. 1a). Mean PPD was 5.8 ± 2.1 mm (range 3–11 mm). Gingival masks of persistently pliable silicon (Frasaco, Tettnang, Germany) were fixed so that the operators could not lift the mask during the trials and visually inspect the furcation or subgingival root area. To approximate a clinical situation as closely as possible to a clinical situation, the study participants also received a panorama image of the study model (digital modified according the contrast for simulation of the alveolar bone and tooth density) and an overview of the PPD in millimeters.

All six multirooted teeth with through-and-through FI (the first and second molars on both sides in the upper jaw (3-rooted molars) and the left first and second molars in the lower jaw (2-rooted molars)) were cleaned by each operator with each instrument, yielding a total of 252 test teeth cleaned by the seven participants (Fig. 1a). The test teeth are modified replicas of extracted human teeth, which are by a three-dimensional CBCT image (OP300 Maxio, 8.7 s, FOV: 5 × 5 cm, Endo Mode, 8.0 mA, 90 kV, 596 mGycm^2^, voxel size: 85 µm, KaVo Dental GmbH, Biberach, Germany) was taken. The acquired DICOM data were used for raw data segmentation to create an accurate three-dimensional digital reconstruction of the teeth (InVesalius 3.1.1, Center for Information Technology Renato Archer, Campinas, Brazil) [17, 18]. Tooth surface and root canal structures were selected as regions of interest (ROIs), and a range of 226 to 1900 Hounsfield units was defined. Using CAD software (Autodesk Meshmixer, Autodesk Direct Limited, Hampshire, United Kingdom) and in vivo data on molar furcation morphologies [19–21], 3D composite replicas were designed and printed with a layer thickness of 25 µm, resulting in an appropriate surface roughness. Using a stereolithography 3D printer (Form 2, Formlabs, Somerville, MA, USA) and a liquid photopolymer resin (White Resin V04 (RS-F2-GPWH-04), Formlabs, Somerville, MA, USA), it was possible to print reproducible geometries with high accuracy [22]. The stereolithographic printing method resulted in different resolutions between the X- and Y-axes on the one hand and the Z-axis on the other hand. In the Z-axis, a maximum roughness of 25 µm was achieved. Depending on the geometry of the object, the roughness in the X- and Y-axes varied between 8 and 25 µm (Fig. 1a). These settings were considered when the objects were aligned for printing in order to achieve the highest possible accuracy. All 3-rooted test teeth were divided vertically into three parts (2-rooted test teeth into two parts), allowing the respective roots to be examined individually for reproducible planimetric evaluation (Fig. 1a).

The test teeth were coated with a thin layer of transparent fluorescent varnish (Shiny White–Rival de Loop Young, Berlin, Germany) between the artificial cementoenamel junction and the alveolar bone, including the entire furcation area (FA), to simulate adherent biofilm. The coating was applied by a reproducible and standardized dipping procedure to achieve consistent thickness for the applied layers of varnish [23]. The varnish fluoresced bright blue when exposed to ultraviolet light (Fig. 1b). The varnish on the root surfaces was placed at a distance of at least 2 mm from the bottom of the pocket and at the marginal edge of the gingiva to simulate pathological circumstances as realistically as possible. All furcation entrances allowed access to the furcation dome with a specially designed probe (Nabers PQ2N, Hu-Friedy Europe, Leimen, Germany), but the furcation entrances were located below the gumline to impede access to the furcation entrance, these lay subgingival.

The artificial biofilms, test teeth and gingival masks were replaced after each instrument.

### Planimetric evaluation

The effectiveness of instrumentation was assessed planimetrically (Fig. 1b). The instrumented roots of the test teeth as well as the test teeth in their entirety were mounted on prepared plastic units (Lego GmbH, Grasbrunn, Germany) individually fitted to each of the six teeth and each root of each tooth. The plastic units with the mounted teeth were then fixed on a camera table in a reproducible position. The teeth and separated roots were irradiated with ultraviolet light (UV-A, 350–370 nm), and one image of the inner surface of each root (3-rooted molars: mesial-buccal, distal-buccal and palatal roots; 2-rooted molars: mesial and distal roots) and one image of the furcation roof of each test tooth (3 images for each of the two 2-rooted teeth and 4 images for each of the four 3-rooted teeth, for a total of n = 22 per utilized instrument and operator) were taken using a camera with a 60-mm macro zoom lens (Canon EOS 500D, Tokyo, Japan) (Fig. 1b). Based on the images, an evaluation of the cleaned surface area was then performed using digital image subtraction (ImageJ, NIH, Bethesda, USA) to calculate relative cleaning effectiveness (RCE, in %) in the removal of simulated subgingival biofilms.

### Outcomes

As a primary outcome, the percentage of removed artificial biofilm RCE was determined. Secondary outcome was the treatment time.

*Relative cleaning efficacy of simulated biofilm removal—RCE in %* RCE was measured as the difference in the area of the simulated biofilm before and after the different root surface areas in the furcation of each test tooth (n = 22) were cleaned by each operator (n = 7); the results were separated for the six types of instrumentation (LAPA-1 with supra/-subgingival nozzle, LAPA-2 with supra/-subgingival nozzle, US, and AIR).

*Treatment time in minutes* Accordingly, the time taken to treat each test tooth with each category of instrument was measured separately for each operator. The time spent changing instruments, etc., was not taken into account.

The investigator (H.B.) was blinded to the instruments and operators when performing the planimetric evaluation.

### Statistical analysis

After sample size calculation using data from a comparable investigation [23], we found n = 42 test teeth per operator to be sufficient to detect an RCE difference of less than 5% between the groups of instruments (power of 80%).

A statistical analysis of the measurements was performed with statistical software (SPSS Statistics 20, IBM, Chicago, IL, USA). The normality of the distribution was confirmed using the Shapiro–Wilk test. Differences between experience groups were analyzed using Kruskal–Wallis nonparametric ANOVA. Post hoc tests were performed using the Mann–Whitney U test, with a Bonferroni correction to adjust for the effects of multiple testing. For pairwise comparisons, the Wilcoxon signed-rank test was used. All tests were two-sided; statistical significance was assumed if *p* ≤ 0.05.

## Results

In total, 252 test teeth (100%) could be analyzed for RCE (different surfaces: n = 924). We found that cleaning the entire FA (all vertical root surfaces and the furcation roof) with SC devices (AIR or US) achieved significantly higher RCE values (42.84 ± 24.24%) than AP with LAPA-1 or LAPA-2 (25.58 ± 26.14%; *p* < 0.001). After dividing the furcation area into the furcation roof and vertical root surface for analysis, we found similar results favoring SC (furcation roof SC/AP: 36.98 ± 26.91%/23.78 ± 28.07%, *p* < 0.001; vertical root surfaces SC/AP: 44.87 ± 25.42%/26.43 ± 26.60%, *p* < 0.001).

These overall results consider only the two overarching groups, SC and AP, and show a difference only between these groups; the intragroup analysis can be found in Tables 1 and 2.

**Table 1:** Overview according to treatment time per tooth and RCE (relative cleaning efficacy) with pairwise instrument comparisons

Instrument	Overall RCE in % (mean ± SD)	RCE for furcation roof in % (mean ± SD)	RCE for all vertical root surfaces in % (mean ± SD)	TrT in s per tooth	Pairwise instrument comparison	*p*-value for RCE*—*overall	*p*-value for RCE*—*furcation roof surfaces	*p*-value for RCE*—*vertical root surfaces	*p*-value for TrT
LAPA-1 with supragingival nozzle	27.05 ± 28.40	24.44 ± 29.69	28.15 ± 29.00	129.67 ± 60.92	Versus LAPA-1 with subgingival nozzle	***p* < 0.001**	***p* < 0.001**	***p* < 0.001**	***p* < 0.001**
					Versus US	***p* < 0.001**	*p* = 0.001	***p* < 0.001**	***p* < 0.001**
					Versus AIR	***p* < 0.001**	***p* < 0.001**	***p* < 0.001**	***p* < 0.001**
LAPA-2 with supragingival nozzle	20.13 ± 22.14	17.15 ± 23.00	21.24 ± 22.73	145.57 ± 76.42	Versus LAPA-1 with supragingival nozzle	*p* > 0.05	*p* > 0.05	*p* > 0.05	*p* > 0.05
					Versus LAPA-1 with subgingival nozzle	***p* < 0.001**	***p* < 0.001**	***p* < 0.001**	*p* = 0.006
					Versus US	***p* < 0.001**	***p* < 0.001**	***p* < 0.001**	***p* < 0.001**
					Versus AIR	***p* < 0.001**	***p* < 0.001**	***p* < 0.001**	***p* < 0.001**
LAPA-1 with subgingival nozzle	41.07 ± 28.95	41.48 ± 30.67	41.34 ± 29.93	213.52 ± 70.95	Versus US	*p* > 0.05	*p* > 0.05	*p* > 0.05	*p* = 0.034
					Versus AIR	*p* > 0.05	*p* > 0.05	*p* > 0.05	*p* > 0.05
LAPA-2 with subgingival nozzle	14.06 ± 14.29	12.04 ± 17.81	14.99 ± 14.33	261.67 ± 100.02	Versus LAPA-2 with supragingival nozzle	*p* > 0.05	*p* > 0.05	*p* > 0.05	***p* < 0.001**
					Versus LAPA-1 with supragingival nozzle	*p* > 0.05	*p* > 0.05	*p* = 0.004	***p* < 0.001**
					Versus LAPA-1 with subgingival nozzle	*p* = 0.002	***p* < 0.001**	***p* < 0.001**	*p* > 0.05
					Versus US	***p* < 0.001**	***p* < 0.001**	***p* < 0.001**	*p* > 0.05
					Versus AIR	***p* < 0.001**	***p* < 0.001**	***p* < 0.001**	*p* > 0.05
US	39.63 ± 22.02	32.54 ± 24.87	41.95 ± 23.53	299.40 ± 120.69	Versus AIR	*p* > 0.05	*p* > 0.05	*p* > 0.05	*p* > 0.05
AIR	46.04 ± 25.96	41.42 ± 28.20	47.78 ± 26.94	274.67 ± 95.80					

Treatment time per tooth (TrT) in seconds and RCE (relative cleaning efficacy) were assessed overall (all surfaces of the furcation area), specifically on the furcation roof and solely on the vertical root surfaces

Average values and *p*-values of RCE were calculated over an N of 252 for test teeth and an N of 924 for different surfaces. All significant* p*-values are highlighted with bold font

**Table 2:** Overview according to RCE (relative cleaning efficacy) in 3-rooted and 2-rooted molars with pairwise instrument comparisons

Instrument	Overall RCE in %	RCE for furcation roof in %	RCE for all vertical root surfaces in %	TrT in s per tooth	Pairwise instrument comparison	*p*-value for RCE*—*overall	*p*-value for RCE*—*furcation roof surfaces	*p*-value for RCE*—*vertical root surfaces	*p*-value for TrT
*3-rooted molars*									
LAPA-1 with supragingival nozzle	15.86 ± 17.75	13.86 ± 19.24	16.55 ± 17.97	130.57 ± 57.99	Versus LAPA-1 with subgingival nozzle	***p* < 0.001**	***p* < 0.001**	***p* < 0.001**	***p* = 0.002**
					Versus US	***p* < 0.001**	***p* < 0.001**	***p* < 0.0013**	***p* < 0.001**
					Versus AIR	***p* < 0.001**	***p* < 0.001**	***p* < 0.001**	***p* < 0.001**
LAPA-2 with supragingival nozzle	13.29 ± 16.20	10.94 ± 16.30	14.04 ± 16.66	142.57 ± 54.33	Versus LAPA-1 with supragingival nozzle	*p* > 0.05	*p* > 0.05	*p* > 0.05	*p* = 1.000
					Versus LAPA-1 with subgingival nozzle	***p* < 0.001**	***p* < 0.001**	***p* < 0.001**	***p* = 0.011**
					versus US	***p* < 0.001**	***p* < 0.001**	***p* < 0.001**	***p* < 0.001**
					Versus AIR	***p* < 0.001**	***p* < 0.001**	***p* < 0.001**	***p* < 0.001**
LAPA-1 with subgingival nozzle	29.60 ± 20.99	32.25 ± 25.63	29.00 ± 21.49	220.29 ± 58.82	Versus US	*p* > 0.05	*p* > 0.05	*p* > 0.05	*p* = 0.190
					Versus AIR	*p* > 0.05	*p* > 0.05	*p* > 0.05	*p* = 0.694
LAPA-2 with subgingival nozzle	11.22 ± 12.48	9.83 ± 16.69	11.91 ± 12.63	267.82 ± 111.41	Versus LAPA-2 with supragingival nozzle	*p* > 0.05	*p* > 0.05	*p* > 0.05	***p* < 0.001**
					Versus LAPA-1 with supragingival nozzle	*p* > 0.05	*p* > 0.05	*p* > 0.05	***p* < 0.001**
					Versus LAPA-1 with subgingival nozzle	***p* < 0.001**	***p* < 0.001**	***p* < 0.001**	*p* = 1.000
					Versus US	***p* < 0.001**	***p* < 0.001**	***p* < 0.001**	*p* = 1.000
					Versus AIR	***p* < 0.001**	***p* < 0.001**	***p* < 0.001**	*p* = 1.000
US	33.44 ± 20.72	28.30 ± 23.52	34.82 ± 21.55	317.64 ± 128.28	Versus AIR	*p* > 0.05	*p* > 0.05	*p* > 0.05	*p* = 1.000
AIR	36.71 ± 21.31	32.25 ± 24.94	36.77 ± 21.78	289.07 ± 107.40					
*2-rooted molars*									
LAPA-1 with supragingival nozzle	56.88 ± 30.07	52.67 ± 34.14	59.07 ± 30.31	127.86 ± 68.66	Versus LAPA-1 with subgingival nozzle	*p* > 0.05	*p* > 0.05	*p* > 0.05	*p* = 0.873
					Versus US	*p* > 0.05	***p* = 0.017**	*p* > 0.05	***p* = 0.004**
					Versus AIR	*p* > 0.05	*p* > 0.05	*p* > 0.05	***p* = 0.006**
LAPA-2 with supragingival nozzle	38.38 ± 25.50	33.73 ± 29.46	40.43 ± 25.65	151.57 ± 110.58	Versus LAPA-1 with supragingival nozzle	*p* = 0.030	*p* > 0.05	*p* = 0.035	*p* = 1.000
					Versus LAPA-1 with subgingival nozzle	***p* < 0.001**	***p* < 0.001**	***p* < 0.001**	*p* = 1.000
					Versus US	*p* > 0.05	*p* > 0.05	*p* = 0.036	***p* = 0.029**
					Versus AIR	***p* < 0.001**	***p* < 0.001**	***p* < 0.001**	***p* = 0.045**
LAPA-1 with subgingival nozzle	71.65 ± 24.84	66.11 ± 29.62	74.23 ± 23.84	200.0 ± 91.63	Versus US	*p* > 0.05	*p* > 0.05	*p* > 0.05	*p* = 1.000
					Versus AIR	*p* > 0.05	*p* > 0.05	*p* > 0.05	*p* = 1.000
LAPA-2 with subgingival nozzle	21.62 ± 16.11	17.93 ± 19.52	23.20 ± 15.50	249.36 ± 74.32	Versus LAPA-2 with supragingival nozzle	*p* > 0.05	*p* > 0.05	*p* > 0.05	***p* = 0.038**
					Versus LAPA-1 with supragingival nozzle	***p* < 0.001**	***p* < 0.001**	***p* < 0.001**	***p* = 0.005**
					Versus LAPA-1 with subgingival nozzle	***p* < 0.001**	***p* < 0.001**	***p* < 0.001**	*p* = 1.000
					versus US	***p* < 0.001**	*p* = 0.002	***p* < 0.001**	*p* = 1.000
					Versus AIR	***p* < 0.001**	***p* < 0.001**	***p* < 0.001**	*p* = 1.000
US	56.14 ± 16.22	43.85 ± 25.12	60.98 ± 17.29	262.93 ± 98.05	Versus AIR	*p* > 0.05	*p* = 0.017	*p* > 0.05	*p* = 1.000
AIR	73.57 ± 14.73	65.85 ± 21.01	77.14 ± 14.49	245.86 ± 60.43					

Average values and *p*-values are shown. All significant* p*-values are highlighted with bold font

In detail, AIR showed the highest effectiveness in cleaning, with an RCE of 46.04 ± 25.96%, while LAPA-2 with a subgingival nozzle had the lowest RCE, at 14.06 ± 14.29% (*p* < 0.001). In addition, LAPA-1 with the subgingival nozzle (41.07 ± 28.95%) showed a similar overall RCE to US (39.63 ± 22.02%, *p* > 0.05). An overview according to RCE values is shown in Table 1.

Regarding specific tooth morphology, the RCE of the whole FA was always higher in the 2-rooted molars (53.04 ± 28.45%, *p* < 0.001) than in the 3-rooted molars (23.19 ± 20.98%) regardless of the instrument used; the same was true for the furcation roof (2-rooted/3-rooted molars: 46.69 ± 31.80% vs. 21.24 ± 23.49; *p* < 0.001) and for all vertical root surfaces (2-rooted/3-rooted molars: 55.84 ± 28.80% vs. 23.85 ± 21.41%; *p* < 0.001). A detailed list of the results by instrument is shown in Table 2.

Independent of which instrument was utilized, the molars with the best RCE values overall and for the furcation roof and all vertical root surfaces were those in the lowest range of furcation height, < 2 mm (Table 3; *p* ≤ 0.002). In contrast, the range of probing depth associated with the best cleaning performance was the middle category, 6–8 mm (Table 3; *p* < 0.001).

**Table 3:** RCE (relative cleaning efficacy) categorized according to furcation height (all, $<$ 2 mm, 2–3 mm, $>$ 3 mm) and probing depth (all, 3–5 mm, 6–8 mm, 9–11 mm)

Category	All	Furcation height < 2 mm	Furcation height 2–3 mm	Furcation height > 3 mm	Pairwise comparison	*p*-value
Overall RCE in %	34.11 ± 25.01	34.86 ± 26.23	30.14 ± 26.05	25.81 ± 28.63	$>$ 3 mm versus 2–3 mm	*p* > 0.05
					$>$ 3 mm versus $<$ 2 mm	***p* < 0.001**
					2–3 mm versus $<$ 2 mm	*p* > 0.05
RCE for furcation roof in %	30.51 ± 24.79	27.54 ± 26.36	32.16 ± 28.32	23.83 ± 32.87	$>$ 3 mm versus 2–3 mm	***p* < 0.001**
					$>$ 3 mm versus $<$ 2 mm	*p* = 0.002
					2–3 mm versus $<$ 2 mm	*p* > 0.05
RCE for all vertical root surfaces in %	35.65 ± 26.16	37.82 ± 27.63	29.76 ± 26.17	26.13 ± 28.55	$>$ 3 mm versus 2–3 mm	*p* > 0.05
					$>$ 3 mm versus $<$ 2 mm	***p* < 0.001**
					2–3 mm versus $<$ 2 mm	*p* = 0.002

		Probing depth 3–5 mm	Probing depth 6–8 mm	Probing depth 9–11 mm		
Overall RCE in %	34.11 ± 25.01	32.45 ± 24.43	39.98 ± 29.26	10.62 ± 14.03	9–11 mm versus 3–5 mm	***p* < 0.001**
					9–11 mm versus 6–8 mm	***p* < 0.001**
					3–5 mm versus 6–8 mm	*p* > 0.05
RCE for furcation roof in %	30.51 ± 24.79	24.78 ± 26.30	37.39 ± 31.76	12.81 ± 21.87	9–11 mm versus 3–5 mm	***p* < 0.001**
					9–11 mm versus 6–8 mm	***p* < 0.001**
					3–5 mm versus 6–8 mm	***p* < 0.001**
RCE for all vertical root surfaces in %	35.65 ± 26.16	34.77 ± 24.74	41.20 ± 30.08	10.11 ± 13.10	9–11 mm versus 3–5 mm	***p* < 0.001**
					9–11 mm versus 6–8 mm	***p* < 0.001**
					3–5 mm versus 6–8 mm	*p* > 0.05

Average values and *p*-values are shown. All significant* p*-values are highlighted with bold font

Treatment time per tooth (TrT) showed significant differences between SC (287.04 ± 109.02 s) and AP (187.61 ± 94.20 s; *p* < 0.001), of which the longest TrT was observed for US (299.40 ± 120.69 s), while the shortest (129.67 ± 60.92 s) was observed for LAPA-1 with a supragingival nozzle (*p* < 0.001) (Table 1). Independent of the type of nozzle, no significant difference between the two AP instruments was detectable (LAPA-1/LAPA-2: 171.60 ± 78.10 s/203.62 ± 106.0 s; *p* = 0.171). The mean TrT of 3-rooted molars was 222.70 ± 114.79 s, and that of 2-rooted molars was 206.29 ± 97.87 s (*p* = 0.266). For further details, please see Table 2.

## Discussion

The main purpose of this study was to compare the efficacy of different groups of powered scalers and air polishing devices with low-abrasiveness powders in removing simulated biofilms from the furcation area. For different concepts of ultrasonic and sonic scalers in the SC group, we observed a 1.5- to 1.7-fold increase in RCE over the entire FA, all vertical root surfaces and the furcation roof compared to AP (*p* < 0.001). However, at least one AP device with a subgingival nozzle (LAPA-1) demonstrated an RCE similar to that of SC (41.07 ± 28.95%, *p* > 0.001) and a 1.3-fold shorter TrT (Table 1). The effect of the complex morphology of the FA on the ability to remove biofilms and mineralized deposits has been discussed extensively for several years [21, 24], but the outcomes of nonsurgical periodontal therapy (NSPT) have remained inconclusive to our knowledge [25, 26]. Therefore, many authors recommended NSPT only for teeth with shallow furcation defects [27, 28] and for advanced FI (i.e., degree 3 according to Hamp et al. [16]), NSPT usually leads to further disease progression in the FA [29]. However, regarding tooth loss, the data of a recent systematic review indicate that in advanced FI, NSPT and open-flap debridement may result in approximately the same survival rates as root amputation/resection, root separation or tunneling [30].

**Fig. 2:**
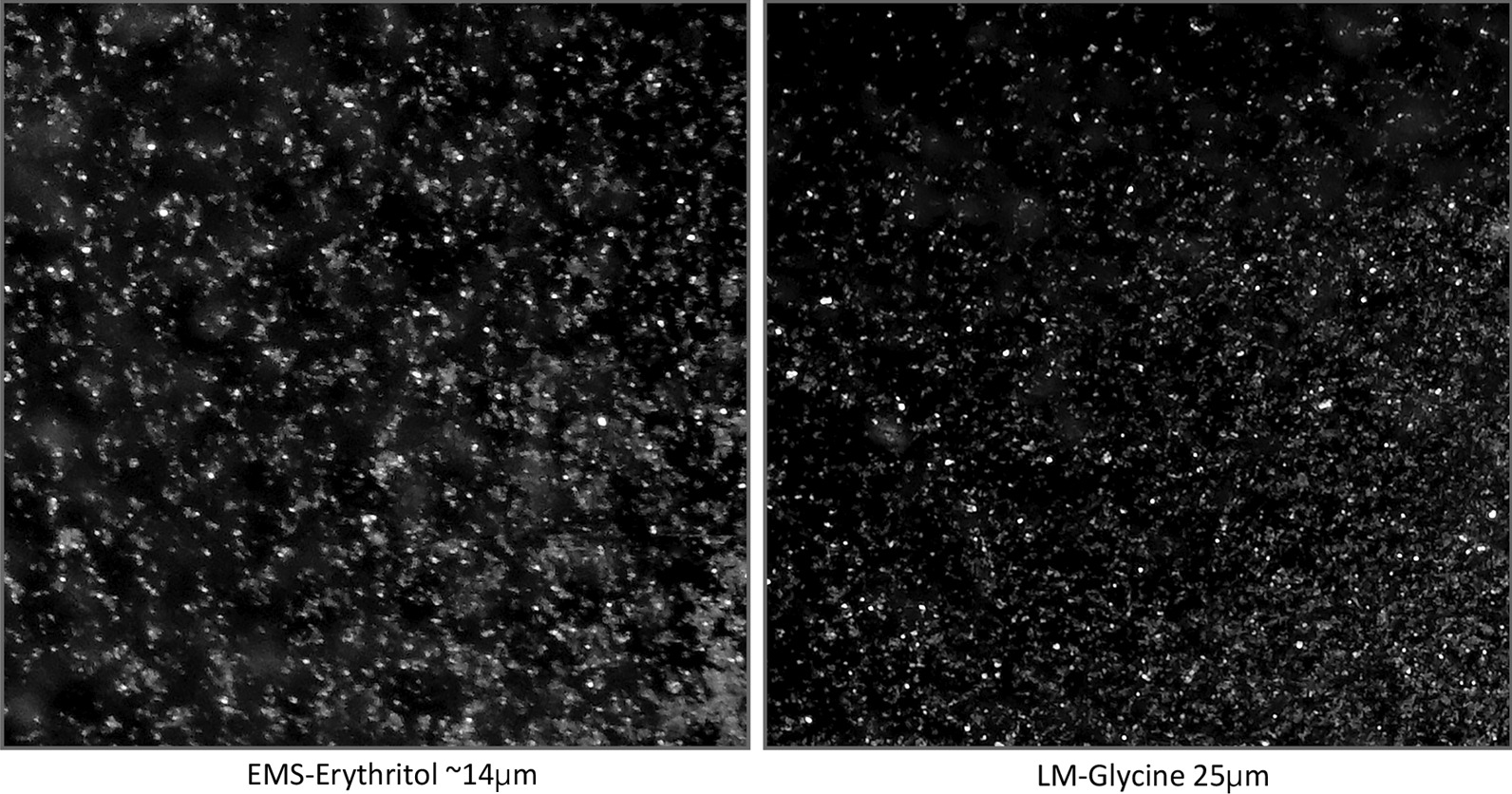
Images of the powders used in the experiment—namely, erythritol (AIR-FLOW PLUS powder, EMS, Nyon, Switzerland) and glycine powders (LM-Glycine Neutral, LM-Instruments Oy, Parainen, Finland) with different particle sizes. The images were taken under an operating microscope (Leica TP 12, Leica Microsystems GmbH, Wetzlar, Germany) at 40 × magnification

**Fig. 3:**
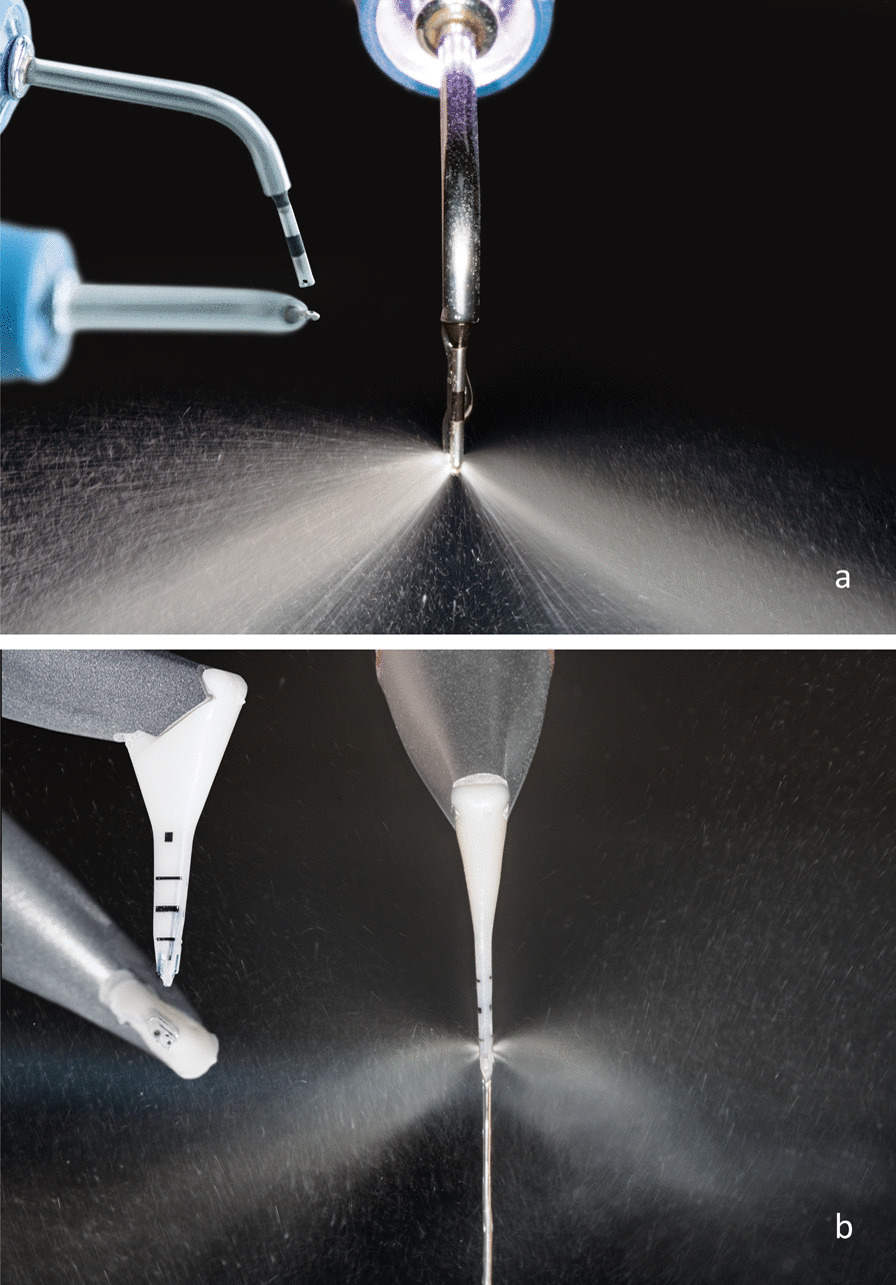
**a** Design and radiation behavior in the detailed images of LAPA-1 with a subgingival instrument tip, used with glycine powder at a particle size of 25 μm (LM-Glycine Neutral, LM-Instruments Oy, Parainen, Finland). **b** Design and radiation behavior in detailed images of LAPA-2 with a subgingival instrument tip, used with erythritol powder at a particle size of 14 µm (PERIOFLOW handpiece, EMS, Nyon, Switzerland)

Despite the deficiencies of in vitro studies such as the current work, we found that a sonic scaler with a slimline tip achieved the highest effectiveness in cleaning the FA, with an RCE of nearly 46%; this percentage does not seem favorable from a clinical point of view, but it is comparable to the results found by other study groups testing sonic or ultrasonic scalers [24, 31]. The results of this in the nineties of the last century published in vitro studies indicated for bud- or ball-shaped scaler tips instead of slimline tips approximately 15% more removed simulated biofilms in the area of furcation. However, we have not tested different scaler tips, as only slimline tips without bud ends were utilized. We assumed that these tips were often used in SPT visits by dental auxiliaries due to availability in practice, whereas the tips with bud ends would need to be purchased separately and incur additional costs. However, a paradigm shift has positioned AP as the perceived standard for SPT [12]. The two tested AP devices utilize glycine (LAPA-1) or erythritol powder (LAPA-2), respectively, with average particle sizes of 25 μm and 14 μm (Fig. 2). The two powders also differ in their chemical nature; for example, glycine is an amino acid that dissolves slowly in water [32], whereas erythritol, a sugar alcohol (polyol) obtained from natural sugar by microbiological fermentation, dissolves comparatively quickly in water. Erythritol is also suitable for patients with diabetes because it has a glycemic factor of 0 [33]. Despite the similar abrasiveness of these two low-abrasiveness powders [34], their different chemical and physical parameters (particle size, distribution pattern, morphology, density, hardness, crystal structure, surface properties, and particle agglomeration) can influence their cleaning performance. Treatment with SPT should not exert any harmful effects on the root surface and should be restricted to the removal of biofilms [8]. With low-abrasiveness powders, AP devices combine minimal abrasion with a maximal cleaning effect [32, 34, 35]. Nevertheless, they largely performed worse than SC in molars with FI in our experimental study, which corroborated the clinical findings of a study by Petersilka et al. [11]. Only with the subgingival nozzle did LAPA-1 show an approximately similar cleaning performance to US (Tables 1, 2); however, LAPA-1 had a nearly 1.4-fold shorter TrT (Table 3, *p* < 0.001). Such a reduction in TrT coupled with high effectiveness in cleaning has already been reported in the literature, e.g., complete biofilm removal on all tooth surfaces of a tooth was observed within 5–10 s at a PPD of less than 5 mm [36]. The cleaning effectiveness decreased to approximately 30% of the cleaning performance of SC at PPD ≥ 5 mm [36]. The tested nozzles for subgingival cleaning were designed in such a way that the powder-water jet was directed vertically onto the root surface to reduce what is known as the "flow pressure" (Fig. 3). This design is thought to contribute to particularly gentle subgingival biofilm removal from the root surface as well as the surrounding soft periodontal tissues, which is important for various reasons such as the prevention of emphysema, but the reduced pressure may also have sacrificed some penetration of the cleaning jet into the FA [34]. This supports our current findings that the specially designed nozzles for subgingival instrumentation perform relatively poorly in the area of the furcation roof, but these nozzles were developed primarily for deep narrow bone pockets and not for the FA [37]. We observed that utilizing the subgingival nozzle in small and narrow areas of the FI is cumbersome; a similar observation was also reported in a split-mouth study by Ulvik et al. [15], as this nozzle tip could not reach the complex horizontal and vertical anatomy of the subgingival furcation and its inherent concavities [37]. In particular, the flexible nozzle of LAPA-2 often bent (Fig. 3), which could be an explanation for the low cleaning efficacy. Additionally, a tight gingiva will further hinder subgingival instrumentation, as we assume based on the use of rubber material to simulate the gingiva [23, 38]. A tight gingiva is often present during SPT; according to the S3 treatment guideline [8], patients with residual probing depths of 4 mm without bleeding on probing after active periodontal treatment should simply proceed to the fourth and final step of therapy. In these patients, it can be assumed that the superficial gingiva is tight and pale pink, possibly even showing physiological stippling, which could further complicate sufficient penetration of the subgingival nozzle of the air polishing devices into the FA.

Therefore, given the results of the current experimental study, it is not surprising that in a retrospective study on the long-term effect of AP in SPT [11], AP with glycine powder was statistically equivalent to SC in single-rooted teeth and teeth without FI. No clinically acceptable results could be obtained for molars with FI. A recent systematic review came to a similar conclusion that AP can achieve equivalent results to those of SC [35]. Petersilka et al. [11] found a negative tendency for the furcation status to deteriorate when AP was used exclusively in SPT. A limiting factor in the evaluation of the results is that the AP group had twice as many smokers and more than twice the number of teeth with FI [11], which are known as risk factors for the possible worsening of periodontal status during SPT [29]. It seems that instrumenting the teeth exclusively with AP does not achieve sufficient cleaning performance in FI. This is also consistent with the fact that in the present experimental study, the best RCE results were obtained in the category with the lowest furcation height (< 2 mm) and at an average probing depth of 6–8 mm. The cleaning performance will be influenced by many parameters (e.g., angle of attack or radiation distance), none of which was recorded in our study. Furthermore, it should be noted that the powders used in this study will interact with our simulated biofilms, and clumping may occur due to a mixture of powder, water and varnish. In particular, glycine powder, which dissolves slowly in water (Fig. 2), could have this tendency [32, 34], and we must assume a negative effect on the RCE of LAPA-2.

Finally, it should be noted that our experimental study did not investigate the effect on either the tooth surface or the soft tissue. A review indicated that regardless of the powder used (glycine or erythritol), there was no significant damage to the gingiva or exposed root surfaces [34, 35]. In contrast, AIR and US can damage the gingiva and tooth surface, but these approaches can also be much more effective than AP in clearing simulated buildup from the surface even in the hard-to-reach FA [11]. Overall, AP devices combine various advantages, such as protection of the surrounding tissue; reduced TrT; high patient acceptance, especially in cases of hypersensitivity; additional antibacterial effects (powder dependent); and reduced noise [10]. Nevertheless, the potential risk of air emphysema should also be taken into account during use [11].

In addition to the details discussed before, our experimental study has further limitations. Due to the character of any in vitro simulation, e.g., using varnish to simulate subgingival biofilms and hard deposits on the root surface of plastic teeth, the results cannot be transferred directly to clinical settings in general [39]. We discussed in detail one of the disadvantages of the gingival masks, namely, the possibility that they limit the ability of the nozzles of the AP devices to penetrate the sulcus; however, the masks will also dampen the vibrations of the oscillating instruments [23, 38–40]. Furthermore, while we considered the type of molar (two- or three-rooted) as a variable, the morphology of the jaw is also likely to influence our results (e.g., restricted access to the FA); however, as the tests were performed similarly and matched exactly for all groups of instruments and operators, we can neglect it.

Overall, the presented in vitro analyses enabled the reproducible investigation of defined parameters that cannot be measured clinically, thus increasing the sensitivity of the comparisons. The authors would like to note that the present experimental study focused on determining the most effective method for cleaning molars with FI by using different instruments to verify the clinical data of the retrospective study by Petersilka et al. [11]. Nevertheless, further studies must be performed in a clinical setting in the future [10].

## Conclusions

Within the limitations of the present experimental study, it can be concluded that cleaning the FA is a complex task in the context of biofilm removal in SPT, which is reflected in the large range of cleaning efficacy results. Overall, our findings corroborated the results of clinical investigations, showing that conventional debridement with sonic or ultrasonic scalers achieves better results but also requires more TrT than the gentler air polishing technique with low-abrasiveness powders. Only one air polishing device (LAPA-1), equipped with a rigid subgingival nozzle, approximately matched the efficacy of an ultrasonic scaler in removing the simulated biofilm from the furcation area. Therefore, future studies should evaluate the influence on soft and hard tissue in long-term SPT.

## Data Availability

The datasets used and analyzed during the current study are not publicly available due to [national data protection law] but are available from the corresponding author on reasonable request.

## Abbreviations

AIR: Sonic scaler
AP: Air polishing
FA: Furcation area
FI: Furcation involvement
LAPA: Low-abrasiveness powder air polishing
NSPT: Nonsurgical periodontal therapy
PPD: Pocket probing depth
RCE: Relative cleaning efficacy
SC: Conventional mechanical debridement
SD: Average values
SPT: Supportive periodontal therapy
TrT: Treatment time
US: Ultrasonic scaler
